# The Cognitive and Behavioural Effects of Perampanel in Children with Neurodevelopmental Disorders: A Systematic Review

**DOI:** 10.3390/jcm13020372

**Published:** 2024-01-10

**Authors:** Giovanna Scorrano, Simona Lattanzi, Vincenzo Salpietro, Cosimo Giannini, Francesco Chiarelli, Sara Matricardi

**Affiliations:** 1 Department of Paediatrics, University of Chieti-Pescara, 66100 Chieti, Italy; giovanna.scorrano@gmail.com (G.S.); cosimo.giannini@unich.it (C.G.); chiarelli@unich.it (F.C.); 2 Department of Experimental and Clinical Medicine, Neurological Clinic, Marche Polytechnic University, 60020 Ancona, Italy; alfierelattanzisimona@gmail.com; 3 Department of Neuromuscular Disease, UCL Institute of Neurology, University College London, London WC1N 3BG, UK; v.salpietro@ucl.ac.uk; 4 Department of Biotechnological and Applied Clinical Sciences (DISCAB), University of L’Aquila, 67100 L’Aquila, Italy

**Keywords:** perampanel, neurodevelopmental disorders, epilepsy, behaviour, cognitive functioning

## Abstract

In children and adolescents with epilepsy, neurodevelopmental comorbidities can impair the quality of life more than seizures. The aim of this review was to evaluate the cognitive and behavioural effects of perampanel (PER) in the paediatric population. We performed a systematic search of the literature, selecting studies published in English including children and adolescents with epilepsy treated with PER. Cognitive and behavioural outcomes were assessed through validated neuropsychological standardised scales. Eighteen studies involving 3563 paediatric patients were included. Perampanel did not impair general cognitive functions and visuospatial skills, whereas a slight improvement in verbal memory and a decline in attentional power were detected. In adolescents with refractory epilepsies, high doses and/or rapid titration of PER and an underlying psychiatric disorder were risk factors for developing or worsening psychiatric outcomes such as anger, aggressiveness, and irritability. Data on children and adolescents treated with new antiseizure medications are scant, and neuropsychiatric effects are tricky to be detected during developmental age. According to the currently available evidence, PER showed an overall favourable risk–benefit profile. Pharmacodynamics, co-administration of other antiseizure medications, and family and personal history of neuropsychiatric disorders should be considered before PER treatment.

## 1. Introduction

Currently, antiseizure medications (ASMs) represent the reference for both the symptomatic treatment of seizures, and the achievement of seizure control, even though about one-third of patients develop drug refractory epilepsy [1,2]. 

Refractoriness to ASMs is a multifactorial condition that appreciably comes to light in childhood and adolescent epilepsies, frequently as part of an underlying neurodevelopmental disorder. Notably, the management of paediatric epilepsies and syndromes is complicated by both more recurrent neuropsychiatric comorbidities and potentially more impactful drug side effects on a developing brain, compared with adult epilepsies [3,4].

In recent years, several new ASMs, including perampanel (PER), emerged in the paediatric epilepsy landscape, with the aim of providing more effective and targeted treatments.

PER is a selective non-competitive α-amino-3-hydroxy-5-methyl-4-isoxazolepropionic acid (AMPA) receptor antagonist, currently approved as a monotherapy or adjunctive treatment in patients aged four years and older with focal seizures with or without secondary generalization, and as adjunctive treatment in patients aged seven years and older with primary generalized tonic–clonic seizures (PGTC) [3,5,6,7,8,9,10]. Previous studies showed the effects of PER on efficacy and tolerability at dosages of 4–12 mg/day, without a significant impairment on general cognitive functions and behaviour [3,6,11]. However, currently available evidence on children and adolescents treated with PER is still limited, and the influence of PER on paediatric epilepsies and syndromes is not entirely defined [10].

The aim of this systematic review is to evaluate the cognitive and behavioural effects of PER in the paediatric population, and to provide a comprehensive overview to guide clinicians in its administration in children and adolescents with epilepsies and neurodevelopmental disorders.

## 2. Materials and Methods

### 2.1. Searching Strategy and Review Organization

This systematic review has been registered in PROSPERO (registration number: CRD42023494687), and reported following the guidelines of the Preferred Reporting Items for Systematic Reviews and Meta-Analyses (PRISMA) statement [12]. A systematic search of the MEDLINE (accessed by PubMed), Cochrane Central Register of Controlled Trials (CENTRAL), and the US National Institutes of Health Clinical Trials Registry (http://www.clinicaltrials.gov (accessed on 15 December 2023)) databases was performed (week 3, December 2023). The search terms were a combination of the following: “epilepsy”, “seizure”, “children”, “adolescent”, “paediatric”, “perampanel”, “cognition”, “attention”, “memory”, “psychiatric”, “behaviour”, and “development”. Details of the search strategies are outlined in Appendix A. The quality of the included studies was assessed using the Newcastle–Ottawa Quality Assessment Scale (NOS). According to this scale, each study has been evaluated on the basis of eight items, described as follows: (1) representativeness of the exposed cohort; (2) selection of the not-exposed cohort; (3) ascertainment of the exposure; (4) demonstration that the outcome of interest was not present at the start of the study; (5) comparability of the cohorts included; (6) assessment of the outcome; (7) adequate length of the follow-up; and (8) adequacy of follow up of cohorts. This score ranges from 0 to 9, and a quality score equal to or higher than three was considered acceptable.

**Figure 1:**
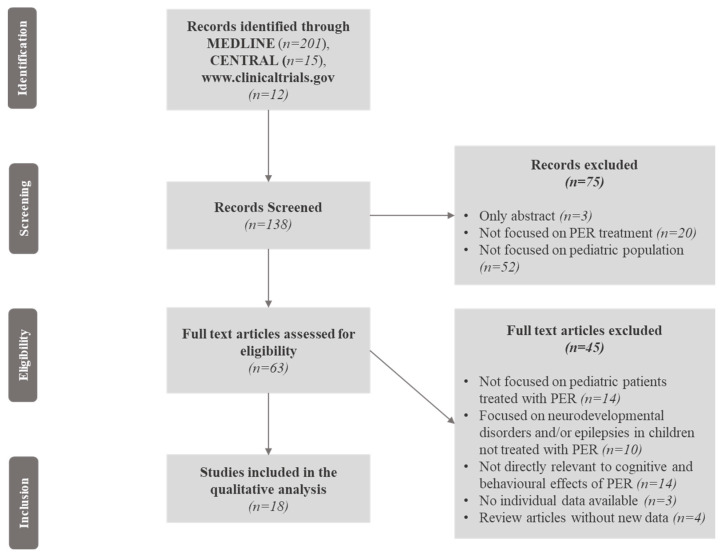
Flow chart of the article screening process. Legend: CENTRAL = Cochrane Central Register of Controlled Trials; PER = perampanel.

We selected studies published in English that included children and adolescents with epilepsy and/or epileptic syndromes who were treated with PER. Cognitive and behavioural outcomes were assessed through objective, validated neuropsychological standardized scales and tests such as intelligence quotient (IQ), the Computerized Assessment System (CDR system) Global Cognition Score, The A-B neuropsychological assessment schedule (ABNAS), the Child Behaviour Checklist (CBCL), and the Lafayette Grooved Pegboard Test (LGPT), exploring global cognitive functions, memory, language, attention, executive functions, visuospatial skills, and behaviour. Neurodevelopmental disorders in paediatric patients treated with PER, such as intellectual disability (ID), autism spectrum disorders (ASDs), and attention deficit hyperactivity disorder (ADHD), were encompassed and investigated as well. The study selection is illustrated in Figure 1, and consisted of several steps where the relevant findings were analysed, with the final inclusion only of studies focused on the cognitive and behavioural effects of PER in paediatric patients with epilepsy associated or not with neurodevelopmental disorders.

### 2.2. Searching Strategy and Review Organization

The collected data included authors, year, study design, sample size and age, PER (dosage and titration strategy) and concomitant ASMs, epilepsy syndrome, neurodevelopmental disorders and neuropsychiatric comorbidities, the effect of PER on global cognitive functioning, the impact of PER on behaviour, attention, aggressiveness, language, memory, mood, executive functions, visuospatial skills, emotion, irritability, and the potential influence of PER on pre-existing neurodevelopmental disorders and psychiatric comorbidities.

## 3. Results

### 3.1. Literature Search

Two hundred and twenty-eight records were initially identified. Of these, 90 records were excluded because only the abstracts were available, the results of clinical trials were not published, or the studies were not focused on paediatric patients; 138 records were, therefore, screened. Of these, seventy-five abstracts were excluded because they were not focused on PER treatment or the paediatric population, and the full texts of 63 articles were reviewed for eligibility. Forty-six articles previously included were excluded (the reasons for exclusion are reported in Figure 1). We finally selected, through the Medline database, eighteen eligible articles involving 3563 paediatric patients after the screening process (Figure 1). A summary of the study quality assessment according to NOS is available in Table S1 of the Supplementary Materials. The included studies comprised seven cohort studies; two retrospective observational studies; two randomized, double-blind, placebo-controlled trials (RCTs), and an extension phase of one of them; three prospective observational studies, two multi-centre observational studies, and one pooled analysis of three clinical trials (Table 1).

### 3.2. Effect of PER on Cognitive Function with Single Domains Analysis

Cognitive disorders are frequent in children and adolescents with epilepsy, and are mainly associated with the underlying aetiology, comorbidities, the epileptic syndrome (e.g., a developmental and epileptic encephalopathy, DEE), ASMs administered, seizure frequency and interictal epileptiform discharges (IEDs) [27]. Dynamic changes in a developing brain affect the cognitive function, especially in children with epilepsy [6,27]. PER did not seem to significantly influence the overall cognitive function in paediatric population with epilepsy associated or not with neurodevelopmental disorders. Specifically, a randomized, double-blind, placebo-controlled, parallel-group phase II study [11], evaluating 123 adolescents (aged 12 to <18 years) with an intelligence quotient (IQ) ≥ 70 and a diagnosis of focal-onset seizures, documented a favourable cognitive profile for PER (add-on therapy, dosage 8–12 mg/day), without significant differences between placebo and PER group, on CDR System Global Cognition Score.

Another multicentre, retrospective, 1 year observational study [15], aiming at characterizing the relationship between PER plasma concentration and cognitive function in 110 adolescents, did not find a relevant effect of PER (dosage 8–12 mg/day) exposure on overall cognitive functioning. Over the years, several studies have come to the same conclusion. Specifically, three observational cohort studies, analysing 14 adolescents (mean age 13.3 years, age range 12.1–14.3 years) [17], 66 children and adolescents (age 14.9 ± 2.3 years) [16] with refractory epilepsy and 2396 adolescents from 45 centres in Europe (95% focal-onset seizures) [18], respectively, showed that PER, administered as add-on therapy, up to a maximum of 12 mg/day, did not confer any significant short- or long-term effects on the global cognition score. Furthermore, a global, multicentre, open-label, single-arm study [13] and a pooled analysis [14] of three clinical trials, involving 180 patients from 4 to 12 years of age with inadequately controlled focal seizures or generalized tonic–clonic seizures, reported that PER (dose range 2–16 mg/day) did not produce any clinically significant effects on global cognitive function. Another multicentre, randomized, double-blind, placebo-controlled study, examining 85 adolescents between 12 and 17 years of age, treated with PER as add-on therapy (dose range 8–12 mg/day), documented a favourable cognitive profile for PER, without significant differences between placebo and PER group, on CDR System Global Cognition Score [22].

Interestingly, two observational cohort studies, analysing 13 patients with Lennox–Gastaut Syndrome (LGS) [28] and 37 adolescents (age 13.78 ± 1.60 years) [29] with focal refractory epilepsy, respectively, documented a slight improvement in cognitive function and performance. Moreover, a quantitative electroencephalogram (qEEG) analysis, performed in a prospective observational study [24], showed a relevant increase in beta1 and total beta bands in children treated with PER (dosage 8 mg/day) as a first add-on treatment, suggesting a beneficial effect of this drug on global cognition.

This evidence suggests how PER treatment did not affect the global cognitive functioning in children who received the drug, who presented a similar cognitive outcome, compared to patients who did not receive PER. Additionally, in multiple studies, a beneficial effect of PER on global cognitive functioning emerged, implying a favourable cognitive profile of the drug. It would be interesting, in the near future, to investigate the molecular mechanisms through which PER acts on cognitive-related pathways, aiming at identifying the underlying cause of the described beneficial effect on cognition.

Concerning the attention domain, in two clinical studies, involving 123 adolescents (aged 12 to <18 years) [11] with an IQ ≥ 70, with a diagnosis of focal-onset seizures, and 114 adolescents [3] treated with PER (8–12 mg/day) as add-on therapy, a worsening in continuity and power of attention, respectively, was detected. On the other hand, two studies [15,29] showed that PER did not significantly influence attention, while Liguori et al. [24] documented a positive effect of PER on the same domain. Data available on attention domain documented, therefore, how there was a slight worsening of attention power, whereas data on attention continuity were weak. Moreover, the differences concerning the power of attention between PER and placebo group were small.

Memory has been explored in several studies. In a randomized controlled study [11], 123 adolescents with focal-onset seizures followed up for 19 weeks and treated with PER (8–12 mg/day); an improvement in the quality of episodic memory, with a worsening in speed memory, without influence on working memory, came to light. Concurrently, two studies [23,30] revealed no meaningful effects of PER on working, episodic, visuospatial, and speed memory, in 114 and 37 adolescents, respectively. Additionally, an observational cohort study [18] found memory problems in 1.3% of 2396 adolescents from 45 centres in Europe (95% focal-onset seizures), using PER as add-on therapy. Studies focused on memory domain documented how PER did not worsen memory, and led to an improvement in the quality of episodic memory as emerged consistently in different studies. By contrast, a worsening in speed memory was detected in only one study, and not coherently further addressed.

Executive functions, perception and logical-abstract thinking were not significantly affected by PER treatment as arose in two studies, involving 37 and 46 adolescents, respectively [29,30].

### 3.3. Effect of PER on Behaviour and Psychiatric Status

Psychiatric and behavioural symptoms are increasingly recognized to be impactful and common in children and adolescents with epilepsy, affecting their quality of life more frequently than seizures [4,19,31,32]. Several factors can lead to psychiatric and behavioural problems such as polytherapy, type, dose and titration of ASMs administered, and a pre-existing psychiatric history. Adolescents seem to be the most vulnerable population in this regard. Over the years, researchers explored the effect of PER on behaviour and psychiatric status in children and adolescents. Concerning aggressiveness and irritability, a 12 months cohort study [16] involving 66 children and adolescents (age 14.9 ± 2.3 years) with refractory epilepsy, using PER (dose range 2–8 mg) as add-on therapy, showed that 10.6% of patients manifested irritability and aggression as the common adverse events. Consistently, two other studies [21,25], analysing 149 and 160 adolescents with refractory epilepsy, treated with adjunctive PER (4–12 mg), revealed that this drug did not have significant effects on psychiatric comorbidities and that irritability was the most common symptom reported in 14.1% and 6.5% of patients, respectively. A prospective observational study [30] reported transient irritability in 3 out of 46 adolescents aged 12–18 years with focal and generalized refractory epilepsy already in therapy with one or two ASMs, treated with PER, ranging from 2 to 8 mg, a good tolerability contour in adolescence even in the medium/long term. Interestingly, a retrospective observational cohort study [33] of 87 patients with LGS who received PER as adjunctive treatment documented that 20% of patients experienced irritability and aggression, particularly in association with rapid titration, duration of epilepsy, and numerous previous treatments. Notably, a cohort study [17] involving 14 adolescents (mean age 13.3 years, age range 12.1–14.3 years) with refractory epilepsy and behavioural problems showed an improvement after adjunctive PER (dose range, 4–12 mg/day) administration, concurrently to improved EEG findings. Consistently, a prospective cohort study [28] revealed an improvement in behaviour for seven patients (53.8%), parallel to seizure reduction, but relied on anecdotal observations. Moreover, a cohort study [29] analysing 37 adolescents (age 13.78 ± 1.60 years) with focal refractory epilepsy treated with PER, showed that the emotional and behavioural profile has not changed after PER, and no significant adverse effects on behaviour have been reported. Coherently, another multicentre, randomized, double-blind, placebo-controlled study, analysing 85 adolescents between 12 and 17 years of age, treated with PER as add-on therapy (dose range 8–12 mg/day), documented a lack of any clinically significant impact of PER on any behavioural measures [22].

Depression and mood disorders have been described in two studies [18,25] and appeared in 4.8% of 2396 and in 4.3% of 160 adolescents, respectively, after PER administration. Interestingly, a cohort study [17] documented an improvement in hyperactivity and impulsivity in 6 out of 14 adolescents with pre-existing behavioural problems treated with PER.

Psychosis/hallucination/delusion (1.8%), anxiety (1.6%), sleep disturbance (1.5%), and suicidal thoughts/ideation (0.8%), have also been described in an observational cohort study [18] of 2396 adolescents from 45 centres in Europe (95% focal-onset seizures) using PER as add-on therapy. 

### 3.4. Cognitive and Behavioural Effects of PER in Children with Neurodevelopmental Disorders

Epilepsy in children and adolescents may frequently be part of an underlying neurodevelopmental disorder. Specifically, ID, ASDs, and ADHD represent the most common neurodevelopmental disorders associated with epilepsy in the paediatric population [4,34,35,36,37,38]. Notably, an observational study [20] followed, for 24 months, 19 patients (10 males, 9 females) between 12 and 18 years old, treated with PER in monotherapy or as add-on therapy (up to a maximum of 12 mg/day). This study highlighted the role of PER in ameliorating neuropsychiatric impairments, including behavioural disturbances in ASD, related to the improvement in clinical seizures and frontal IEDs, and unrelated to seizure and EEG improvement in at least some ASD patients, suggesting that PER may be well tolerated as a therapy, even for ASD patients with refractory epilepsy. Additionally, a 24 month retrospective study [26], evaluated the effects of PER on 20 children with ID and epilepsy, treated (dose range 1–12 mg) in monotherapy or as add-on therapy. Behavioural effects were encountered in 40.3% of patients and the most frequent were aggression, agitated behaviour, disruptive behaviour, and mood symptoms. Interestingly, a trend has been highlighted, indicating that a lower number of concomitant ASMs was associated with more behavioural adverse effects. Notably, this study showed that pre-existing behavioural problems or polypharmacy did not predict the occurrence of additional behavioural adverse effects in children with ID and epilepsy.

## 4. Discussion

The prescription of some ASMs can be influenced by psychiatric and behavioural effects, which could occur even after weeks or months the initiation of the treatment, presumably related to an individual susceptibility due to an underlying neuropsychiatric disorder [39]. Predisposing endogenous factors have been individuated and include, pre-existing psychiatric disorders, frontal lobe epilepsy, absence epilepsy, and/or refractory epilepsy [40].

Notably, data on children and adolescents treated with new ASMs are scant, and neuropsychiatric effects are tricky to detect during developmental age [4,41,42]. Over the years, cognitive, psychiatric, and behavioural effects have been detected in patients treated with PER. Among glutamate receptors, AMPA receptors are the main inducers of glutamate-related excitatory neurotransmission, with a pivotal role in synaptic homeostasis and plasticity during neurodevelopmental stages [43].

As a non-competitive AMPA antagonist, PER is not overcome by increased glutamate concentrations at the synapsis. This condition has been associated with the onset of psychiatric and behavioural reactions such as impulsive aggression, presumably mediated by stimulation of glutamate receptors in the amygdala, hypothalamus, and periaqueductal grey matter. Even though genetic manipulation of AMPA receptors in mice models has been associated with behaviour disorders, the glutamate influence on behaviour appears tricky, with improvement and/or worsening of neuropsychiatric symptoms induced by AMPA block, at different doses [39]. Consistently, irritability and aggressiveness are the most frequent behaviour effects of PER in the paediatric population, and they are influenced by the PER dosage administered and the titration schedule. These symptoms mainly affect adolescents who appear vulnerable, especially if they present a pre-existing psychiatric comorbidity. Nevertheless, a worsening of pre-existing behavioural symptoms such as aggressiveness and/or irritability has not been significantly described in the paediatric population to date. Actually, one study [17] revealed an improvement in adolescents with behavioural problems concurrently to ameliorated EEG findings. Furthermore, patients who experienced irritability and aggressiveness had been exposed to high doses, rapid titration of PER, numerous previous treatments, and a long-lasting epilepsy [33]. Therefore, patients with pre-existing psychiatric comorbidities and long-lasting epilepsy treated with polytherapy should be carefully evaluated and monitored, avoiding high doses and rapid titration of PER.

Depression and mood disorders have been reported at a lower rate. Similarly, recognising these symptoms as PER-related, rather than associated with a multifactorial aetiology, is challenging [6,44]. However, they should be appraised before PER administration, to prevent their potential worsening.

Cognitive disorders are frequent in children and adolescents with epilepsy, and are related to several causes. Further cognitive impairment due to ASMs appears arduous to detect, especially in patients with pre-existing neurodevelopmental disorders [4].

Systematic data currently available on the effect of PER on cognition functioning in paediatric patients with epilepsy revealed how PER presents a favourable cognitive profile, leading to a constant slight improvement in global cognition functioning and episodic memory and a small worsening in power of attention. Animal models and/or human organoids could clarify the underlying molecular mechanisms related to the single-domain analysis effects described in the literature. The current evidence on cognition functioning suggests, therefore, that PER is a promising, effective and tolerated drug, suggesting that its use might be encouraged, especially in school-age patients. Furthermore, in children with refractory epilepsy, the simultaneous use of different ASM mechanisms of action associated with polytherapy represents the major potential risk factor for cognitive impairment [4]. Therefore, rational polytherapy should also take into account pre-existing cognitive impairment when prescribing add-on PER [45]. 

Epilepsy in children and adolescents may frequently be part of an underlying neurodevelopmental disorder, with complex clinical presentation and management. Patients with neurodevelopmental disorders frequently present refractory epilepsy and require polytherapy with ASMs, potentially associated with an increased risk of cognitive and behavioural effects. Currently, few studies have examined the neuropsychiatric effects of PER in children and adolescents with both epilepsy and neurodevelopmental disorders. The current evidence [20] indicates that PER is well tolerated as a therapy, even for ASD patients with refractory epilepsy, leading to an improvement in neuropsychiatric impairments, including behavioural problems. Concurrently, children with ID and epilepsy treated with PER manifested behavioural effects such as aggressiveness, irritability, and mood disorders. Notably, pre-existing behavioural problems or polypharmacy did not predict the occurrence of additional behavioural adverse effects [26].

Clinical studies concerning children with ADHD and epilepsy treated with PER are not available. The current evidence suggests that patients with pre-existing behavioural problems should be worth considering before PER administration, avoiding a rapid titration and a high dosage [46]. Further, PER has been associated with improvement in hyperactivity and impulsivity in 42.8% of patients with pre-existing behavioural problems and refractory epilepsy [17], suggesting that it might be contemplated in patients with ADHD, when clinically indicated.

This review of clinical studies concerning the use of PER in the paediatric population indicated that it has an overall favourable risk–benefit profile, especially in adolescents. However, there is a lack of evidence of data on children under twelve years of age, and further studies could be useful to improve the current knowledge on the use of PER in younger patients, especially during the first years of neurodevelopment.

Moreover, a personalized approach is crucial, focusing on the familial and personal history of the child, so as to analyse the individual susceptibility to cognitive and behavioural drug effects. In the near future, both the growing number of paediatric patients treated with PER and molecular studies better clarify the influence of PER on cognitive function and behaviour at the central nervous system level, and the PER complex profile. 

## Figures and Tables

**Table 1:** Overview of studies exploring cognitive and behavioural effects of perampanel (PER) in the paediatric population.

N	Authors, Year	Type of Study	Population and Neurodevelopmental Disorder Examined	Age Group	Treatment	Cognitive and Behavioural Effects	Main Findings
1	Meador et al., 2016 [11]	Randomized, double-blind, placebo-controlled, parallel-group phase II study 19-week follow-up	123 adolescents (12 to <18 years) with an IQ ≥ 70 and a diagnosis of focal-onset seizures	Adolescents aged 12 to 18 years	79 patients treated with PER, 44 with placebo PER: 8–12 mg/day 1–2 other ASMs	CDR System Global Cognition Score (overall *p* > 0.05): Power of attention (*p* > 0.05) Working memory (*p* > 0.05) Quality of episodic memory ↑ (*p* = 0.012) Continuity of attention ↓ (*p* = 0.013) Speed of memory ↓ (*p* = 0.032) Letter fluency (*p* > 0.05) Category fluency (*p* > 0.05) LGPT (*p* > 0.05)	Worse performance under PER in continuity of attention and speed of memory. Better performance in quality of episodic memory. Favourable cognitive profile for PER
2	Villanueva et al., 2016 [13]	Multicentre, retrospective, 1-year observational study aiming at characterizing the PK profile of PER and the relationship between PER plasma concentration and cognitive function	110 adolescents with the same characteristics as in Meador et al., 2016 [11]	Adolescents aged 12 to 18 years	Same as in Meador et al., 2016 [11]	CDR System Global Cognition Score (overall *p* > 0.05): Power of attention (*p* > 0.05) Working memory (*p* > 0.05) NS Quality of episodic memory ↑ (*p* < 0.05) Continuity of attention ↓ (*p* < 0.05) Speed of memory (*p* > 0.05) NS	No significant relationship between PER exposure and overall cognitive function
3	Auvin et al., 2017 [14]	Prospective cohort study Mean follow-up duration: 10.8 months (range, 1–24 months)	13 patients with LGS. Mean age 12.8 years (median 13, range 6–18.5) Patients had received prior treatment with 6–9 different ASMs	Patients aged 6 to 18.5 years	PER was initiated at 2 mg/day and titrated to a median maximum dose of 6 mg/day (range 4–8)	Parents and physicians reported improvement in cognitive function and/or behaviour for 7 patients (53.8%) parallel to seizure reduction	No formal conclusions on cognitive function and behaviour, as they were not formally assessed but relied on anecdotal observations
4	Piña-Garza et al., 2018 [3]	Open-label extension phase of the trial by Meador et al., 2016 [11]	Patients who completed all scheduled visits in the double-blind phase in the study by Meador et al., 2016 [11] were eligible (114 adolescents)	Adolescents aged 12 to 18 years	Same as Meador et al., 2016 [11]; those assigned to placebo switched to PER 2 mg/day, which was up- titrated weekly in 2 mg increments, up to a maximum of 12 mg/day	CDR System Global Cognition Score (overall *p* > 0.05): Power of attention ↓ (*p* = 0.03) Working memory (*p* > 0.05) NS Quality of episodic memory (*p* > 0.05) NS Continuity of attention (*p* > 0.05) Speed of memory *p* > 0.05) Letter fluency (*p* > 0.05) Category fluency (*p* > 0.05) Lafayette Grooved Pegboard test (*p* > 0.05)	PER did not significantly affect cognitive parameters, except for the power of attention. No clinically meaningful effects on growth and development
5	Rohracher et al., 2018 [15]	Observational cohort study, 12 months	2396 adolescents from 45 centres in Europe (95% focal-onset seizures), using PER as add-on	Adolescents aged 12 to 18 years	PER as add-on: 2 mg/day, which was up- titrated weekly in 2-mg increments, up to a maximum of 12 mg/day	No significant relationship between PER exposure and overall cognitive function. Depressed mood and mood disorders (4.8%), Mental confusion/slowing/psychomotor retardation (2.3%), Psychosis/hallucination/delusion (1.8%), Anxiety (1.6%), Sleep disturbance (1.5%), Memory problems (1.3%), Other psychiatric TEAEs, Suicidal thoughts/ideation (0.8%), Speech problems/slurred speech (0.9%)	1-year retention rate of 48%, seizure free in 9%; TEAEs were in line with previous reports (68%)
6	Lin et al., 2018 [16]	Cohort study, 12 months	66 children and adolescents (age 14.9 ± 2.3 years) with refractory epilepsy, using PER as add-on	Patients aged 14.9 ± 2.3 years	The targeted doses varied depending on clinical response and tolerability. The mean maximal dosage of add-on PER was 4.9 ± 2.9 mg (median 4 mg, range: 2–8 mg).	No significant relationship between PER exposure and overall cognitive function. The appearance of irritability and aggression were only founded in seven patients (10.6%)	TEAEs in 35.7%, leading to discontinuation of PER in 12.1%. Irritability (10.6%) was the most common
7	Fogarasi et al., 2020 [17]	Global, multicentre, open-label, single-arm study To evaluate the effects of PER on cognitive function, secondary safety endpoints included changes from base line in ABNAS at week 23	180 patients from 4 to 12 years of age	Patients aged 4 to 12 years	Mean PER dose 7.0 mg/day (range 2–16 mg/day) PER used as oral suspension (0.5 mg/mL)	No significant changes in clinics at week 23 from baseline as assessed by ABNAS in total score and each of the domains	PER did not produce any clinically significant effects on cognitive function, secondary function at week 23 safety endpoints compared with baseline
8	Majid et al., 2016 [18]	Report aiming at exploring the PER exposure–response relationships for cognition and safety with data from Fogarasi et al., 2020 [17]	156 PER-treated subjects aged 4 to <12 years with partial-onset seizures or primary generalized tonic–clonic seizures	Patients aged 4 to 12 years	Same as in Fogarasi et al., 2020 [17]	No discernible relationship between PER and changes from baseline for ABNAS, CBCL, or LGPT	Cognitive function is not clinically impaired by PER administration
9	Santamarina et al., 2020 [19]	Cohort study, 12 months	149 adolescents with epilepsy, using PER as add-on	Adolescents aged 12 to 18 years	The mean dose of PER among patients receiving PER at 12 months was 6.2 mg/day (median 6 mg/day)	Concomitant ASMs did not affect the tolerability of PER in the current study and adding PER did not seem to have significant effects on psychiatric comorbidities. Irritability was the most common AE leading to discontinuation	TEAEs in 48.3%, leading to PER discontinuation in 10.1%. Dizziness (15.4%), irritability (14.1%), and drowsiness (14.1%), were the most common
10	Kanemura et al., 2020 [20]	Cohort study, 12 months	14 adolescents (mean age 13.3 years, age range 12.1–14.3 years) with LEV-resistant epilepsy and behavioural problems, using PER in polytherapy	Adolescents aged 12.1 to 14.3 years	Mean dose of PER was 9.43 mg/day (range, 4–12 mg/day)	Hyperactivity and impulsivity improved in 6 patients. PER does not confer any significant short- or long-term effects on the global cognition score in adolescent patients	PER treatment may be effective in decreasing behavioural problems in association with improved EEG findings
11	Moraes et al., 2020 [21]	Nested cohort study, 12 months	160 adolescents with refractory focal epilepsy using PER as monotherapy or add-on	Adolescents aged 12 to 18 years	Titration was in 2 mg increments to reach a target dose (4–12 mg) and occurred weekly to monthly depending on specialist preference and patient tolerability	Improved QOL, mood, and irritability following introduction and maintenance for at least six months on PER	In the retrospective cohort, mood changes (7.6%), depression (4.3%), and irritability (6.5%) were the most common
12	Operto et al., 2020 [22]	Cohort study, 12 months	37 adolescents (age 13.78 ± 1.60 years) with focal refractory epilepsy	Adolescents aged 13.78 ± 1.60 years	The mean dose of PER at T1 and T2 follow up was 3.13 ± 0.83 mg/d and 3.50 ± 0.86 mg/d, respectively	PER therapy did not significantly influence attention and executive functions; on contrary, it was possible to highlight a slight improvement in cognitive performance. Even the emotional and behavioural profile has not changed after PER, and no significant adverse effects on behaviour have been reported	No negative effect on executive function, emotion, or behaviour with PER, but 5 patients (13.5%) discontinued PER due to dizziness and headache
13	Operto et al., 2021 [23]	Prospective observational study, 12 months	46 adolescents aged 12–18 years with focal and generalized refractory epilepsy already in therapy with one or two ASMs	Adolescents aged 12 to 18 years	Patients received a variable dose of PER ranging from 2 to 8 mg (mean dose = 3.40 ± 1.17)	Visuospatial memory and perception were not significantly affected by PER therapy. These results, therefore, suggest that PER has a good tolerability contour in adolescence even in the medium/long term	No significant TEAEs were reported, with the exception of transient irritability (n = 3) and dizziness (n = 2), which did not require drug withdrawal
14	Liguori et al., 2021 [24]	Prospective observational study	10 children treated with PER as first add-on treatment	Children aged 11.20 ± 5.70 years	The initial titration of PER was 2 mg daily for 2 weeks, then followed by the increment to 4 to 8 mg daily until the first follow-up visit at 3 months	A significant increase in beta1 and total beta bands were found in children suggesting a beneficial effect of this drug on cognition and alertness, although the sample size was small	Positive effect of PER on attention and cognition in patients with epilepsy. PER does not worsen daytime sleepiness at 3 months
15	Matricardi et al., 2023 [25]	Retrospective observational cohort study	87 patients with LGS who received PER as adjunctive treatment	Patients aged < 18 years	The titration of PER was ≤2 mg every week in 20 (23.0%) patients, 2 mg every 2 weeks in 52 (59.7%), and 2 mg every 3–4 weeks in 15 (17.3%) patients. The maxi-mum dose given was 6 (IQR = 4–8) mg/day	20% of the patients experienced irritability and aggression, particularly in association with rapid titration, duration of epilepsy, and numerous previous treatments.	In approximately three quarters of patients who experienced TEAEs, PER was withdrawn, whereas TEAEs were no longer detectable following down-titration in the remaining cases. The inverse association between the occurrence of TEAEs and the time to seizure relapse among responders may be due to the reactive reduction in PER dosage to improve treatment tolerability. PER appears to be effective in patients with LGS and generally well tolerated, with a possible partial loss of efficacy in a few patients over time
16	Kanemura et al., 2021 [20]	Observational Study, 24 months	19 patients (10 males, 9 females) between 12 and 18 years old treated with PER in monotherapy or as add-on therapy Neurodevelopmental disorder: ASD	Patients aged 12 to 18 years	After a 3 month baseline period, PER was initiated once daily, starting at a dose of 2 mg/day for the first 2 weeks, and with dosage increasing in increments of 2 mg/day every 2 weeks. PER dosage was adjusted up to a maximum of 12 mg/day, based on the judgment of the clinicians	Utility of PER in improving neuropsychiatric impairments including behavioural disturbances in ASD related to improvement in clinical seizures/frontal IEDs, and unrelated to seizure/EEG improvement in at least some ASD patients. PER may be well tolerated as a therapy even for ASD patients with intractable epilepsy.	Utility of PER in behavioural improvements for some, but not all, ASD patients. Treatment with PER appears well tolerated
17	Snoeijen-Schouwenaars et al., 2017 [26]	Retrospective study, 24 months	20 children treated with PER in monotherapy or as add-on therapy Neurodevelopmental disorder: ID	Patients aged < 18 years	The initial PER dose ranged from 0.5 mg to 2 mg once a day. The titration rate was guided individually by the treating neurologist. The mean maximum daily dosage PMP was 5.6 mg (range 1–12 mg)	Behavioural adverse effects were encountered in 40.3% of patients. The most common behavioural adverse effects were aggression, agitated behaviour, disruptive behaviour, and mood symptoms. There was a trend indicating that a lower number of concomitant ASMs was associated with more behavioural adverse effects. Pre-existing behavioural problems or polypharmacy did not predict the occurrence of additional behavioural adverse effects	Side effects were encountered in nearly 60% of our patients which is in line with both the previous studies that did not focus on patients with ID.
18	Lagae et al., 2016 [13]	Multicenter, randomized, double-blind, placebo-controlled, parallel-group study	85 adolescents between 12 and 17 years of age, with an intelligence quotient (IQ) score ≥ 70, and refractory focal-onset seizures, treated with PER as add-on therapy	Adolescents aged 12 to 17 years	PER as add-on therapy: 2 mg/day uptitrated weekly in 2-mg increments to a target dose range of 8–12 mg/day.	Cognitive Drug Research (CDR) System has been shown not to be significantly different from that of placebo. PER did not have a detrimental effect on “competence” or “problem” scores compared with placebo. Lack of any clinically significant impact of PER on any behavioural measures.	The most frequently reported AEs (≥10%) being dizziness, somnolence, and headache, with an overall safety profile similar to previous studies

CDR system = computerized assessment system; ABNAS = The A-B neuropsychological assessment schedule; CBCL = Child Behaviour Checklist; LGPT = Lafayette Grooved Pegboard Test; PER = Perampanel; TEAE = treatment-emergent adverse event; ASM = antiseizure medication; LGS= Lennox–Gastaut Syndrome; ASD = autism spectrum disorder, IED = interictal epileptiform discharge; ID = intellectual disability; EEG = electroencephalogram; QOL = quality of life; N = number.

## Data Availability

Not applicable.

## Supplementary Materials

The following supporting information can be downloaded at: https://www.mdpi.com/article/10.3390/jcm13020372/s1, Table S1: Newcastle-Ottawa Quality Assessment Scale single paper score.

## Appendix A

The Medline full search strategy was (“epilep*”[All Fields] OR “seizure*”[All Fields]) AND (“perampanel”[Supplementary Concept] OR “perampanel”[All Fields]) AND (“child”[MeSH Terms] OR “child”[All Fields] OR “children”[All Fields] OR “child s”[All Fields] OR “children s”[All Fields] OR “childrens”[All Fields] OR “childs”[All Fields] OR “adolescen*”[All Fields] OR “pediatr*”[All Fields] OR “paediatr*”[All Fields]) AND (“cognit*”[All Fields] OR “intellect*”[All Fields] OR “attent*”[All Fields] OR “memor*”[All Fields] OR “psych*”[All Fields] OR “behav*”[All Fields] OR “develop*”[All Fields] OR “neurodevelop*”[All Fields]).

Concerning clinicaltrials.gov, the search strategy was epilep* OR seizure* | children OR adolescen* OR pediatr* OR paediatr* | perampanel | Interventional Studies | Studies with Results. Of 7 of the 12 articles selected through this search strategy on clinicaltrials.gov, results have been published and related reports are online available (ClinicalTrials.gov ID NCT00700310, NCT00699582, NCT02849626, NCT01393743, NCT00735397, NCT00368472, and NCT01527006).

The CENTRAL search strategy was (epilep* OR seizure*) AND (perampanel) in Title, Abstract, Keywords + children OR adolescen* OR pediatr* OR paediatr*).

## References

[1] Kwan P., Arzimanoglou A., Berg A.T., Brodie M.J., Allen Hauser W., Mathern G., Moshé S.L., Perucca E., Wiebe S., French J. (2010). Definition of drug resistant epilepsy: Consensus proposal by the ad hoc Task Force of the ILAE Commission on Therapeutic Strategies. Epilepsia.

[2] Wang Y., Chen Z. (2019). An update for epilepsy research and antiepileptic drug development: Toward precise circuit therapy. Pharmacol. Ther..

[3] Piña-Garza J.E., Lagae L., Villanueva V., Ben Renfroe J., Laurenza A., Williams B., Kumar D., Meador K.J. (2018). Long-term effects of adjunctive perampanel on cognition in adolescents with partial seizures. Epilepsy Behav..

[4] Moavero R., Santarone M.E., Galasso C., Curatolo P. (2017). Cognitive and behavioral effects of new antiepileptic drugs in pediatric epilepsy. Brain Dev..

[5] Pascarella A., Iannone L.F., Di Gennaro G., D’Aniello A., Ferlazzo E., Gagliostro N., Ursini F., Bonanni P., Paciello N., Romigi A. (2020). The efficacy of perampanel as adjunctive therapy in drug-resistant focal epilepsy in a “real world” context: Focus on temporal lobe epilepsy. J. Neurol. Sci..

[6] Fong Y.-O., Huang P., Hsu C.Y., Yang Y.-H. (2022). Effects of Perampanel on Seizure Control, Cognition, Behavior, and Psychological Status in Patients With Epilepsy: A Systematic Review. J. Clin. Neurol..

[7] Liguori C., Manfredi N., Renna R., Izzi F., Pagliuca M., Pagliuca F., Mercuri N.B., Fabio P. (2020). Comparison of the effectiveness and tolerability of perampanel and brivaracetam: A real-world, observational, retrospective study. Epileptic Disord..

[8] Lee S.-A., Jeon J.Y., Kim H.-W. (2020). Effect of perampanel on aggression in patients with refractory focal epilepsy: A 6-month longitudinal study. Epilepsy Behav..

[9] Hanada T., Hashizume Y., Tokuhara N., Takenaka O., Kohmura N., Ogasawara A., Hatakeyama S., Ohgoh M., Ueno M., Nishizawa Y. (2011). Perampanel: A novel, orally active, noncompetitive AMPA-receptor antagonist that reduces seizure activity in rodent models of epilepsy: Perampanel: A Novel AMPA-R Antagonist. Epilepsia.

[10] Sun S., Li X., Liu X. (2023). Efficacy, tolerability and safety of perampanel in children and adolescents with epilepsy: Systematic review and meta-analysis. Brain Dev..

[11] Meador K.J., Yang H., Piña-Garza J.E., Laurenza A., Kumar D., Wesnes K.A. (2016). Cognitive effects of adjunctive perampanel for partial-onset seizures: A randomized trial. Epilepsia.

[12] Moher D., Shamseer L., Clarke M., Ghersi D., Liberati A., Petticrew M., Shekelle P., Stewart L.A., PRISMA-P Group (2015). Preferred reporting items for systematic review and meta-analysis protocols (Prisma-P) 2015 statement. Syst. Rev..

[13] Lagae L., Villanueva V., Meador K.J., Bagul M., Laurenza A., Kumar D., Yang H. (2016). Adjunctive perampanel in adolescents with inadequately controlled partial-onset seizures: A randomized study evaluating behavior, efficacy, and safety. Epilepsia.

[14] Auvin S., Dozieres B., Ilea A., Delanoë C. (2017). Use of perampanel in children and adolescents with Lennox–Gastaut Syndrome. Epilepsy Behav..

[15] Rohracher A., Zimmermann G., Villanueva V., Garamendi I., Sander J.W., Wehner T., Shankar R., Ben-Menachem E., Brodie M.J., Pensel M.C. (2018). Perampanel in routine clinical use across Europe: Pooled, multicenter, observational data. Epilepsia.

[16] Auvin S. (2022). Paediatric epilepsy and cognition. Dev. Med. Child Neurol..

[17] Fogarasi A., Flamini R., Milh M., Phillips S., Yoshitomi S., Patten A., Takase T., Laurenza A., Ngo L.Y. (2020). Open-label study to investigate the safety and efficacy of adjunctive perampanel in pediatric patients (4 to <12 years) with inadequately controlled focal seizures or generalized tonic-clonic seizures. Epilepsia.

[18] Majid O., Laurenza A., Ferry J., Hussein Z. (2016). Impact of perampanel on pharmacokinetics of concomitant antiepileptics in patients with partial-onset seizures: Pooled analysis of clinical trials. Br. J. Clin. Pharmacol..

[19] Santamarina E., Bertol V., Garayoa V., García-Gomara M.J., Garamendi-Ruiz I., Giner P., Aranzábal I., Piera A., Arcos C., Esteve P. (2020). Efficacy and tolerability of perampanel as a first add-on therapy with different anti-seizure drugs. Seizure.

[20] Kanemura H., Sano F., Hoshino H., Takayama K., Aihara M. (2020). Effects of perampanel on secondary bilateral synchrony and behavioral problems in adolescents with epilepsy showing insufficient response with levetiracetam. Seizure.

[21] Moraes J.S., Hepworth G., Ignatiadis S., Dharan A., Carne R., Seneviratne U., Cook M.J., D’Souza W.J. (2020). Improved irritability, mood, and quality of life following introduction of perampanel as late adjunctive treatment for epilepsy. Epilepsy Behav..

[22] Operto F.F., Pastorino G.M.G., Mazza R., Di Bonaventura C., Matricardi S., Verrotti A., Carotenuto M., Viggiano A., Coppola G., Elia M. (2020). Perampanel tolerability in children and adolescents with focal epilepsy: Effects on behavior and executive functions. Epilepsy Behav..

[23] Operto F.F., Vivenzio V., Scuoppo C., Padovano C., Roccella M., Quatrosi G., Pastorino G.M.G. (2021). Perampanel and Visuospatial Skills in Children With Epilepsy. Front. Neurol..

[24] Villanueva V., Montoya J., Castillo A., Mauri-Llerda J., Giner P., López-González F.J., Piera A., Villanueva-Hernández P., Bertol V., Garcia-Escrivá A. (2018). Perampanel in routine clinical use in idiopathic generalized epilepsy: The 12-month GENERAL study. Epilepsia.

[25] Matricardi S., Cesaroni E., Bonanni P., Foschi N., D′Aniello A., Di Gennaro G., Striano P., Cappanera S., Siliquini S., Freri E. (2023). Long-term effectiveness of add-on perampanel in patients with Lennox–Gastaut syndrome: A multicenter retrospective study. Epilepsia.

[26] Snoeijen-Schouwenaars F.M., van Ool J.S., Tan I.Y., Schelhaas H.J., Majoie M.H. (2017). Evaluation of perampanel in patients with intellectual disability and epilepsy. Epilepsy Behav..

[27] Lin K.-L., Lin J.-J., Chou M.-L., Hung P.-C., Hsieh M.-Y., Chou I.-J., Lim S.-N., Wu T., Wang H.-S. (2018). Efficacy and tolerability of perampanel in children and adolescents with pharmacoresistant epilepsy: The first real-world evaluation in Asian pediatric neurology clinics. Epilepsy Behav..

[28] Liguori C., Spanetta M., Izzi F., Russo A., Guerra A., Mercuri N.B., Placidi F. (2020). Perampanel Increases Cortical EEG Fast Activity in Child and Adult Patients Affected by Epilepsy: A Quantitative EEG Study. Clin. EEG Neurosci..

[29] Piña-Garza J.E., Rosenfeld W., Saeki K., Villanueva V., Yoshinaga H., Patten A., Williams B., Malhotra M. (2020). Efficacy and safety of adjunctive perampanel in adolescent patients with epilepsy: Post hoc analysis of six randomized studies. Epilepsy Behav..

[30] Mammì A., Ferlazzo E., Gasparini S., Bova V., Neri S., Labate A., Mastroianni G., Bianco C.L., Cianci V., Aguglia U. (2022). Psychiatric and Behavioural Side Effects Associated With Perampanel in Patients With Temporal Lobe Epilepsy. A Real-World Experience. Front. Neurol..

[31] Goji H., Kanemoto K. (2019). The effect of perampanel on aggression and depression in patients with epilepsy: A short-term prospective study. Seizure.

[32] Toledo M., Gonzalez-Cuevas M., Miró-Lladó J., Molins-Albanell A., Falip M., Martinez A.B., Fernandez S., Quintana M., Cambrodi R., Santamarina E. (2016). Sleep quality and daytime sleepiness in patients treated with adjunctive perampanel for focal seizures. Epilepsy Behav..

[33] Dolton E., Choudry A. (2014). Perampanel and Challenging Behaviour in Intellectual Disability and Epilepsy: A Management Dilemma. Case Rep. Psychiatry.

[34] Robertson J., Hatton C., Emerson E., Baines S. (2015). Prevalence of epilepsy among people with intellectual disabilities: A systematic review. Seizure.

[35] Lee B.H., Smith T., Paciorkowski A.R. (2015). Autism spectrum disorder and epilepsy: Disorders with a shared biology. Epilepsy Behav..

[36] Reilly C., Atkinson P., Das K.B., Chin R.F., Aylett S.E., Burch V., Gillberg C., Scott R.C., Neville B.G. (2015). Features of autism spectrum disorder (ASD) in childhood epilepsy: A population-based study. Epilepsy Behav..

[37] Parisi P., Moavero R., Verrotti A., Curatolo P. (2010). Attention deficit hyperactivity disorder in children with epilepsy. Brain Dev..

[38] Kanemura H., Sano F., Hoshino H., Aihara M. (2021). Efficacy of perampanel in epilepsy patients with autism spectrum disorder. Epilepsy Res..

[39] Hansen C.C., Ljung H., Brodtkorb E., Reimers A. (2018). Mechanisms Underlying Aggressive Behavior Induced by Antiepileptic Drugs: Focus on Topiramate, Levetiracetam, and Perampanel. Behav. Neurol..

[40] Chen B., Detyniecki K., Choi H., Hirsch L., Katz A., Legge A., Wong R., Jiang A., Buchsbaum R., Farooque P. (2017). Psychiatric and behavioral side effects of anti-epileptic drugs in adolescents and children with epilepsy. Eur. J. Paediatr. Neurol..

[41] Mula M., Cavalheiro E., Guekht A., Kanner A.M., Lee H.W., Özkara Ç, Thomson A., Wilson S.J. (2017). Educational needs of epileptologists regarding psychiatric comorbidities of the epilepsies: A descriptive quantitative survey. Epileptic Disord..

[42] Guerrini R. (2006). Epilepsy in children. Lancet.

[43] Perversi F., Costa C., Labate A., Lattanzi S., Liguori C., Maschio M., Meletti S., Nobili L., Operto F.F., Romigi A. (2023). The broad-spectrum activity of perampanel: State of the art and future perspective of AMPA antagonism beyond epilepsy. Front. Neurol..

[44] Stephen L.J., Wishart A., Brodie M.J. (2017). Psychiatric side effects and antiepileptic drugs: Observations from prospective audits. Epilepsy Behav..

[45] Fattorusso A., Matricardi S., Mencaroni E., Dell’Isola G.B., Di Cara G., Striano P., Verrotti A. (2021). The Pharmacoresistant Epilepsy: An Overview on Existant and New Emerging Therapies. Front. Neurol..

[46] Verrotti A., Moavero R., Panzarino G., Di Paolantonio C., Rizzo R., Curatolo P. (2018). The Challenge of Pharmacotherapy in Children and Adolescents with Epilepsy-ADHD Comorbidity. Clin. Drug Investig..

