# Supplementary material for: The Cognitive and Behavioural Effects of Perampanel in Children with Neurodevelopmental Disorders: A Systematic Review
Source: J Clin Med. 2024 Jan 10;13(2):372. doi: 10.3390/jcm13020372 (PMC10816822; doi:10.3390/jcm13020372)
Supplement: Supplementary file 1 [file jcm-13-00372-s001.zip › jcm-2806198-supplementary.pdf]

Reference	Representativeness of the exposed cohort	Selection of the non exposed cohort	Ascertainment of exposure	Demonstration that outcome of interest was not present at start of study	Comparability of the cohorts included	Assessment of outcome	Was follow-up long enough for outcomes to occur?	Adequacy of follow up of cohorts	Total score
Meador et al, 2016	*	*	*	*	*	*	*	-	*******
Villanueva et al., 2016	*	-	*	*	-	*	*	-	*****
Auvin et al., 2017	*	-	*	*	-	*	*	-	*****
Piña-Garza et al., 2018	*	*	*	*	*	*	*	-	*******
Rohracher et al, 2018	*	-	*	*	-	*	*	-	*****
Lin et al, 2018	*	-	*	*	-	*	*	-	*****
Fogarasi et al., 2020	*	-	*	*	-	*	-	-	****
Majid et al., 2016	*	-	*	*	-	*	-	-	****
Santamarina et al., 2020	*	-	*	*	-	*	*	-	*****
Kanemura et al., 2020	*	-	*	*	-	*	*	-	*****
Moraes et al., 2020	*	-	*	*	-	*	*	-	*****
Operto et al., 2020	*	-	*	*	-	*	*	-	*****
Operto et al., 2021	*	-	*	*	-	*	*	-	*****
Liguori et al., 2021	*	-	*	*	-	*	-	-	****

Matricardi et al, 2023	*	-	*	*	-	*	*	-	*****
Kanamura et al., 2021	*	-	*	*	-	*	*	-	*****
Snoeijen-Schouwenaars et al., 2017	*	-	*	*	-	*	*	-	*****
Lagae et al., 2016	*	*	*	*	*	*	*	-	******

Supp.Table S1. Newcastle-Ottawa Quality Assessment Scale single paper score.
